# Association of tooth loss and gallstones: National Health and Nutrition Examination Survey 2017-2018

**DOI:** 10.7150/ijms.98492

**Published:** 2024-07-16

**Authors:** Ziqing Yu, Dongsheng Wu, Gechong Ruan, Xuemin Yan, Yinghao Sun, Wei Han, Xiaoyin Bai, Hong Yang

**Affiliations:** 1 Department of Gastroenterology, Peking Union Medical College Hospital, Chinese Academy of Medical Sciences & Peking Union Medical College, Beijing 100730, China.; 2 Department of Epidemiology and Biostatistics, Peking Union Medical College Hospital, Chinese Academy of Medical Sciences & Peking Union Medical College, Beijing 100730, China.

**Keywords:** gallstones, oral health, mediation, blood glucose

## Abstract

**Introduction:** Gallstones are one of the most common digestive diseases globally, with an estimated affected population of 15% in the United States. Our aim is to assess the current association between oral health and gallstones, exploring potential mediation factors.

**Methods:** Self-reported gallstones were determined based on medical condition questionnaires. Dental status was assessed by dental professionals and oral health questionnaire. Mediation analysis was conducted for body mass index, blood glucose, triglycerides, and cholesterol, and the percentage of mediation effects was calculated.

**Results:** We included 444 patients with gallstones and 3565 non-gallstone participants from National Health and Nutrition Examination Survey. After fully adjusting for all covariates, the prevalence of gallstones is higher when the number of missing teeth is at T3 compared to T1 (odds ratio [OR]: 1.93, confidence interval [CI]: 1.14 - 3.26, p = 0.02, p-trend = 0.01), and there was an inverted L-shaped association between missing teeth and gallstones, with an inflection point of 17. Bone loss around mouth was also associated with gallstones (OR: 1.78, 95% CI: 1.27 - 2.48, p = 0.002), but not root caries and gum disease. Mediation analysis identified blood glucose as a crucial mediator, with a mediation effect ratio of 4.91%.

**Conclusions:** Appropriate lifestyle interventions for patients with missing teeth may help delay the onset of gallstones, such as healthy dietary habits, trace elements supplementing, and managing weight and blood sugar levels. Further exploration of the relationship between oral health and overall health contributes to disease prevention and comprehensive medical management.

## Introduction

Gallstones are one of the most common digestive system diseases globally, imposing a significant healthcare burden in the United States, affecting up to 15% of the population.^1^ Gallstones are primarily classified into cholesterol stones, pigment stones, and mixed stones, with cholesterol stones and the cholesterol component (mainly in mixed stones) constituting over 80% of all stones.^2^ Generally, gallstones do not cause any clinical symptoms, but 10-25% of patients may experience biliary pain, acute cholecystitis, and even serious complications, greatly impacting patients' quality of life.^3^ Preventing and controlling gallstones are particularly important. The risk factors for gallstones are classified into two categories: non-modifiable and modifiable. The former includes age, gender, race, pregnancy, and other conditions such as cirrhosis.^4^, ^5^ The latter encompasses factors like medications, diabetes, metabolic syndrome, and obesity.^4^

A study based on the U.S. population from 1988 to 1994 found a connection between gallstone disease confirmed by ultrasound examination and poor oral health. Missing teeth, considered a crucial indicator of oral health, were identified as an independent predictive factor for gallstone disease.^6^ The association between oral health and gallstones could be influenced by various factors such as body mass index (BMI) and blood lipids. BMI was identified as independent risk factors for gallstone.^7^ The incidence of gallstones significantly increased in obese patients, and mendelian randomization studies confirmed a causal impact of body mass index on gallstone risk.^8^, ^9^ Study had also linked periodontitis, dental caries, and tooth loss to a higher BMI. 10 Additionally, microorganisms associated with periodontitis, such as actinomycetes, had been correlated with waist circumference, serum high-density lipoprotein cholesterol, and metabolic syndrome.^11^ However, existing studies on the association between missing teeth and gallstones used outdated data (1988-1994) and did not reflect the current relationship between oral hygiene and gallstones. Moreover, the mediating effects of obesity, blood sugar, and blood lipids on the association between oral health and gallstones have not been considered. Therefore, we aim to explore the association between missing teeth and self-reported gallstones in the general non-institutionalized U.S. population using data from the National Health and Nutrition Examination Survey (NHANES) and investigate the mediating effects of obesity and blood sugar and lipids. This aims to better elucidate the impact of oral health on gallstone disease across the entire population.

## Materials and Methods

### Study Design and Population

**Figure 1:**
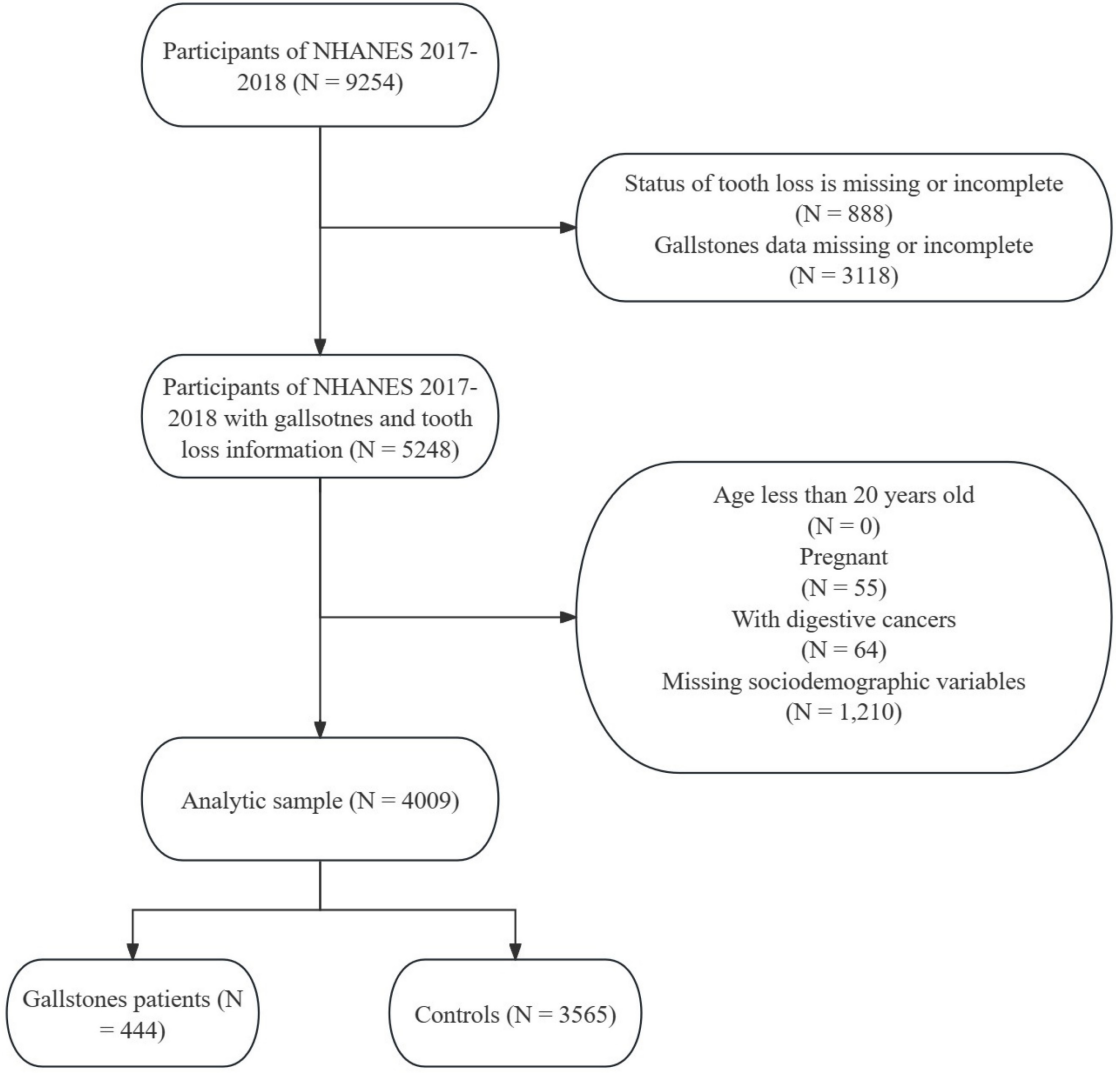
** Flow chart of participants' selection and composition.** NHANES: National Health and Nutrition Examination Survey

NHANES is a nationally representative cross-sectional survey designed by the National Center for Health Statistics in the United States, utilizing a complex multistage sampling design. Its purpose is to assess the health and nutritional status of non-institutionalized civilian residents in the U.S. All data and guidelines are publicly available through https://www.cdc.gov/nchs/nhanes/index.htm. NHANES data has been released every two years since 1999, with individual medical histories and medication usage collected through questionnaire surveys, and laboratory data tested using standard methods. Research for each NHANES cycle had received approval from the National Center for Health Statistics Research Ethics Review Board. Ethical approval documents are available on the official website (https://www.cdc.gov/nchs/nhanes/irba98.htm?s_cid=qr2022). All participants had provided written informed consent. The medical health questionnaire contains specific questions ("Has a doctor or other health professional ever told that had gallstones?") to determine if participants have gallstones. Dental professionals assess tooth loss, all of whom are licensed dentists in at least one U.S. state. Another health technician assists in directly inputting the observer's findings into the computer data collection system. All oral health assessments take place in a mobile examination center equipped with a portable dental chair, lighting, and compressed air. Dental examiners undergo initial training (including lectures, model reviews, simulated exercises, and standardized courses) and on-site training (including standardization and calibration). Additionally, a reference examiner visits each dental examiner 2-3 times a year, conducting about 20 repeat examinations during each visit to assess inter-examiner consistency. We additionally included bone loss around the mouth, root caries, and gum disease for sensitivity analysis. The oral health questionnaire contains information about bone loss around the mouth and gum disease, while the data for root caries is consistent with tooth loss.^12^ Participants with missing tooth and gallstone information, digestive system cancer, under 20 years old, and with missing covariates were excluded (Figure 1). Additionally, considering that pregnancy, as a special condition, has a significant impact on gallstones and oral health, we referred to previous studies and excluded it as well.^13^-^15^ We ultimately included 444 cases of gallstone patients for analysis from NHANES 2017-2018, representing a population of 23,835,653 self-reported gallstone individuals in the United States. The control group consists of 3,565 non-gallstone participants from the NHANES 2017-2018 database, representing a general non-institutionalized population of 185,294,082 in the United States.

### Sociodemographic Variables

We analyzed a wide range of sociodemographic characteristics using the extensive NHANES questionnaire and laboratory data. Age (age in years of the participant at the time of screening), sex, race (White, Black, and Other), marital status (married/with partner and alone), education level (highest grade or level of school, classified as ≤Highschool and >Highschool), and family monthly poverty level ("a ratio of monthly family income to the Health and Human Services' poverty guidelines specific to family size") were obtained from the demographic data (DEMO_J) of NHANES 2017-2018. While smoking status (never, former, now) and alcohol consumption (No or Yes) were obtained from smoking - cigarette use (SMQ_J) and alcohol use questionnaire (ALQ_J), respectively. BMI, which was calculated by weight and height from weight history questionnaire (WHQ_J), was categorized as underweight/normal weight (<25 kg/m²), overweight (25-29.9 kg/m²), and obese (≥30 kg/m²). The diagnosis of diabetes followed the Global guideline for type 2 diabetes or was based on information from a doctor and the use of diabetes-related medications.^16^ High blood pressure was defined as taking antihypertensive medications, being informed by a doctor of hypertension, or having a systolic blood pressure ≥130 mmHg or diastolic blood pressure ≥80 mmHg.^17^ Milk consumption (never, rarely, sometimes, often) was obtained from the Diet Behavior & Nutrition questionnaire regarding milk use in the past 30 days. Vigorous physical activity was derived from the Physical Activity Questionnaire and is defined as "vigorous-intensity activity that causes large increases in breathing or heart rate like carrying or lifting heavy loads, digging, or construction work for at least 10 minutes continuously". Depression was assessed by the 9-question Patient Health Questionnaire (PHQ-9) and classified into five categories based on PHQ-9 scores (0-4, 5-9, 10-14, 15-19, and 20-27). Additionally, dietary fiber, total fat, and total cholesterol intake were obtained from the 24-hour dietary recall questionnaire. Laboratory test data, including blood glucose, lipids, cholesterol, etc., were collected through standard monitoring procedures.

### Statistical Analysis

We chose special weights for new weight calculations to obtain nationally representative results while conforming to a complex sampling design. Descriptive statistical analysis was used to present the basic characteristics of patients and controls (continuous variables presented as mean ± standard error, categorical variables as numbers and percentages). Weighted logistic regression was employed to test demographic differences between cases and controls. Considering the limitation of the sample size, we selected covariates that are clinically relevant or have been found to have a relatively clear correlation, with a difference in univariate analysis (P < 0.2), to be included in the multivariate analysis.^18^ Tooth loss was classified based on tertiles for stratified analysis, and weighted logistic regression was also used to examine the association between gallstones and missing teeth. Restricted cubic splines were used to assess the potential nonlinear relationship between the number of tooth losses and gallstones. The "mediation" package in the R software was used to analyze the mediating effects of BMI, blood sugar, triglycerides, total cholesterol, and high-density lipoprotein cholesterol on the relationship between the number of tooth losses and gallstones. Mediation analysis was accomplished by establishing three paths, including: (1) from exposure to mediation; (2) from mediation to outcome (direct effect); (3) from exposure to outcome (total effect). The total effect reflects the sum of the direct and mediation (indirect) effects. The percentage of mediation effects was calculated using the following formula: (mediation effect/total effect) × 100. The significance test of the mediation analysis used the Bootstrapping method. All statistical analyses and graphing were performed using R-4.2.2.

### Ethical Statements

Since the data for this study are entirely derived from the NHANES public database, patient personal information is replaced by identification codes ("sequence"), thus obviating the need for ethical approval from the authors' institution. Additionally, written informed consent was obtained from each patient included in the study and the study protocol conforms to the ethical guidelines of the 1975 Declaration of Helsinki as reflected in a priori approval by the Ethics Committee of National Center for Health Statistics Research Ethics Review Board for each NHANES cycle. Ethical approval documents are available on the official website (https://www.cdc.gov/nchs/nhanes/irba98.htm?s_cid=qr2022). All participants had provided written informed consent to National Center for Health Statistics Research Ethics Review Board.

## Results

### Characteristics of the Study Participants

Table 1 displays the basic characteristics of self-reported gallstone patients compared to controls. A total of 444 self-reported gallstone patients were included in the analysis, representing a population of 23,835,653 gallstone individuals in the United States. Among gallstone patients, the average age was 56.51, with females accounting for 74.66%, and a predominance of White (70.01%). Gallstone patients had an average daily intake of 15.66g of dietary fiber, 80.14g of fat, and 263.18mg of cholesterol. Compared to non-gallstone patients, self-reported gallstone patients were older, predominantly female, more likely to smoke but less likely to drink, had a higher BMI, engaged in less physical activity, and were more prone to depression. Regarding comorbidities, gallstone patients had a higher prevalence of diabetes, hypertension, hyperlipidemia, and metabolic syndrome. Additionally, gallstone patients exhibited higher levels of blood glucose and triglycerides.

### Association Between Gallstones and Missing Teeth

After fully adjusting for all covariates, the prevalence of self-reported gallstones was higher when the number of missing teeth is at T3 compared to T1 (odds ratio [OR]: 1.93, confidence interval [CI]: 1.14-3.26, p = 0.02, p -trend = 0.01, Table 2). In the sensitivity analysis, bone loss was significantly associated with gallstones (OR: 1.78, 95% CI: 1.27 - 2.48, p = 0.002), whereas no association was observed for root caries and gum disease, though the OR values for both were greater than one (Table S1). Table 3 presents the results of stratified analysis between missing teeth and self-reported gallstones. After adjusting for sociodemographic characteristics, the prevalence of gallstones increased with the number of missing teeth in the following populations: 20-40 years old (p-trend = 0.02), females (p-trend = 0.005), white individuals (p-trend = 0.02), other races (p-trend = 0.04), >Highschool education (p-trend = 0.003), married or with partner (p-trend = 0.02), high family income (p-trend = 0.01), non-smokers (p-trend = 0.01), smokers (p-trend = 0.01), non-drinkers (p-trend = 0.003), and obese individuals (p-trend = 0.01).

**Figure 2:**
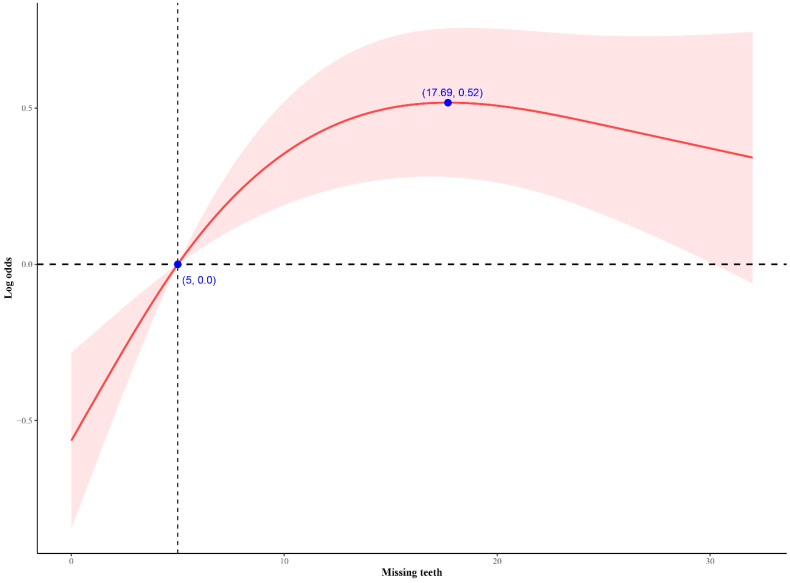
** Association between missing teeth and self-reported gallstones.** The number of missing teeth showed a non-linear relationship with self-reported gallstones (non-linear P value < 0.0001). When the number of missing teeth is less than 17, the prevalence of gallstone disease increases with an increasing number of missing teeth.

Figure 2 shows the nonlinear association between the number of missing teeth and gallstones. In the restricted cubic splines analysis, the relationship between the number of missing teeth and self-reported gallstones demonstrated an approximately inverted L-shaped curve (non-linear p-value < 0.0001). When the number of missing teeth was less than 17, the prevalence of gallstones increases with an increase in the number of missing teeth.

### Mediation analysis

After adjusting for sociodemographic variables, BMI, blood glucose, and triglycerides were significantly associated with missing teeth and self-reported gallstones. Table 4 displays the results of the mediation analysis. In the overall association between missing teeth and gallstones, the mediation proportions for BMI, blood glucose, triglycerides, total cholesterol, and high-density lipoprotein cholesterol were 1.59%, 4.91%, 3.05%, 0.9, and 11.97%, respectively.

## Discussion

In this large cross-sectional study, we found that individuals with a higher number of missing teeth or with bone loss around mouth had a higher prevalence of self-reported gallstones. The prevalence of gallstones increased rapidly with the number of missing teeth when the count was below 17. Meanwhile, blood glucose was identified as an important mediating factor.

The mutual association between oral health and gallstones may be related to common influencing factors such as dietary nutrition and micronutrients. A higher dietary inflammatory index and greater pro-inflammatory potential were associated with an increased risk of periodontitis and tooth loss.^19^ A study based on NHANES 2005-2008 indicated a positive correlation between the total number of teeth and the intake of proteins, most vitamins, and minerals, and a negative correlation with carbohydrate intake. Moreover, a healthy eating index was inversely proportional to the number of missing teeth.^20^ This suggests that tooth loss is associated with lower dietary quality and reduced nutrient intake. Fermentable carbohydrates (sugar and starch) are common dietary risk factors for periodontal disease and dental caries, while deficiencies in micronutrients such as vitamin C, vitamin D, or vitamin B12 may be related to the occurrence and development of both diseases.^21^ Specifically, a diet high in sugar, saturated fat, low polyols, low fiber, and low polyunsaturated fat increased the risk of periodontal disease. Conversely, a diet low in sugar, high in fiber, and with a high omega-6 to omega-3 fatty acid ratio reduced the risk of periodontal disease.^22^ Similar dietary components also have a similar impact on the formation of gallstones. High intake of refined sugar and sweets, high fructose intake, low fiber content, high fat, and low vitamin C increased the risk of gallstone formation, while high intake of monounsaturated fat and fiber, olive oil, fish (omega-3 fatty acids), plant proteins, fruits, coffee, and vitamin C had protective effects. 23 Vitamin D also plays a role in both gallstones and oral health. A study from NHANES suggested that higher micronutrient intake and higher serum levels of vitamin D seemed to mitigate the burden of chronic oral diseases.^24^ A population study in Copenhagen, focusing on individuals aged 41-71, indicated a correlation between gallstones and low vitamin D exposure in utero, unrelated to serum 25-hydroxyvitamin D levels. 25 This implies a potential genetic causative link between gallstones and oral health. In the sensitivity analysis, we found that only bone loss was significantly associated with self-reported gallstones. Tooth loss and bone loss around mouth are often indicators of long-term oral problems, suggesting more severe oral lesions. Gum disease is more likely to reflect short-term oral health levels and is more likely to be corrected early.^26^ This suggests that more severe poor oral health is closely related to gallstones, and early intervention for minor oral lesions may have a preventive effect on gallstones.

The microbiota simultaneously influences oral health and the calcification of gallstones, possibly representing a potential mechanism linking the two. The mineralization process of dental calculus was similar to other ectopic calcifications like gallstones, and nanobacteria were a major cause of pathogenic calcification, potentially playing a crucial role in the calcification processes of dental and gallstones.^27^ Nanobacteria, later termed calcifying nanoparticles, may be involved in the calcification processes of various conditions such as dental pulp, salivary glands, and renal stones.[28]Additionally, there may be an association between the upper digestive tract microbiota and dental health. A large cohort study found that poor dental health was associated with lower diversity in the upper digestive tract microbiota, while poor periodontal health was associated with higher microbial diversity and potential pathogenic species.^29^ Microbes may serve as a bridge between oral health and biliary diseases, and further research is needed to explore the impact of microbiota on both oral health and biliary diseases.

Obesity and blood sugar may be related factors between oral health and gallstones. Several studies had found a correlation between obesity and gallstones.^7^, ^8^ However, we did not observe a significant mediating effect of BMI between tooth loss and gallstones in this study because tooth loss was not correlated with BMI (p = 0.78) in this study. Tooth loss can occur due to reasons such as trauma, dental caries, periodontal disease, fractured teeth, failed endodontic treatment, etc.^10^ Results showing a positive correlation between BMI and tooth loss primarily come from the Korean national health screening cohort.^30^ However, a study based on the Korean National Health and Nutrition Examination Survey found a correlation between underweight and tooth loss.^31^ The relationship between missing teeth and BMI may be more complex. An Iranian study suggested that although the periodontal health of overweight and obese individuals was far worse than that of normal-weight individuals, oral health was not influenced by body mass index.^32^ Nonlinear relationships and the influence of age and gender may be part of the complex association between oral health and BMI. A cross-sectional study involving more Korean adults confirmed that there was a "J-shaped" relationship between BMI and poorer self-rated oral health, especially in underweight men and obese women. Moreover, in men and women over 65, underweight and obesity were associated with poorer self-rated oral health.^33^ Blood sugar may have a great effect on the relationship between gallstones and oral health. The European Prospective Investigation into Cancer and Nutrition study found that gallstone was a risk factor for type 2 diabetes.^34^ Mendelian randomization study also confirmed an independent causal relationship between type 2 diabetes and gallstones.^35^ Diabetic patients had a higher risk of tooth loss and dental caries, and in the United States, one in every five cases of tooth loss was related to diabetes.^21^, ^36^ Diabetes also has an unpredictable impact on the relationship between diet and gallstones. More prospective research is still needed to explore the effects of blood sugar and blood lipids on oral health and gallstones.

Cross-sectional study design inevitably affects the accuracy of causal inference. Due to limitations in NHANES data, we did not incorporate more oral health-related variables such as the severity of periodontitis, dental caries, and low-density lipoprotein for analysis. The definition of gallstones relied on patient self-reported questionnaires rather than ultrasound. This leads to the omission of the vast majority of asymptomatic gallstone patients, resulting in selection bias. Nevertheless, our study has several strengths. To our knowledge, this is the first article assessing the relationship between missing teeth and self-reported gallstones in the general U.S. population. We also identified a non-linear relationship between missing teeth and gallstones. Blood glucose emerged as important mediating factors between oral health and gallstones. Based on the NHANES design, the results can be generalized to the non-institutionalized U.S. population, approximately 208,446,109 people.

In conclusion, we found a higher risk of self-reported gallstones with more missing teeth, and the risk of gallstones increased with the number of missing teeth up to 17. Blood glucose proved to be an important mediating factor between tooth loss and gallstones. Implementing appropriate lifestyle interventions for individuals experiencing tooth loss may mitigate the risk of gallstones, including healthy dietary habits, micronutrient supplements, weight management, and blood glucose control. Additionally, further exploration of the relationship between oral health and overall health contributes to comprehensive health management, personalized healthcare, and disease prevention.

## Supplementary Material

Supplementary table.

## Figures and Tables

**Table 1:** Descriptive characteristics of participants with and without self-reported gallstones

Variable	Total	Controls (n=3565)	Gallstones (n=444)	P value
**Age**	48.20(0.77)	47.13(0.80)	56.51(0.81)	< 0.0001
**Age group**				< 0.0001
20-40	75580617.73(36.14)	71419910.87(38.54)	4160706.85(17.46)	
40-60	73603748.92(35.20)	65403375.53(35.30)	8200373.39(34.40)	
≥60	59945370.24(28.66)	48470796.97(26.16)	11474573.27(48.14)	
**Sex**				< 0.0001
Female	106892632.99(51.11)	89096848.62(48.08)	17795784.38(74.66)	
Male	102237103.90(48.89)	96197234.76(51.92)	6039869.14(25.34)	
**Ethnicity**				0.07
White	133784567.59(63.97)	117096959.62(63.20)	16687607.97(70.01)	
Black	22226806.38(10.63)	20750124.77(11.20)	1476681.62(6.20)	
Other	53118362.91(25.40)	47446998.99(25.61)	5671363.92(23.79)	
**Marital status**				0.09
Alone	79038573.79(37.79)	68486977.84(36.96)	10551595.95(44.27)	
Married/With partner	130091163.10(62.21)	116807105.54(63.04)	13284057.56(55.73)	
**Educational level**				0.74
≤Highschool	79782746.44(38.15)	70398756.39(37.99)	9383990.05(39.37)	
>Highschool	129346990.45(61.85)	114895326.98(62.01)	14451663.47(60.63)	
**Family monthly poverty level**				0.26
≤1.30	48527930.79(23.20)	43389207.63(23.42)	5138723.16(21.56)	
1.30-1.85	27496667.23(13.15)	23485481.97(12.67)	4011185.26(16.83)	
>1.85	133105138.87(63.65)	118419393.78(63.91)	14685745.09(61.61)	
**Smoking status**				0.01
Never	119522153.47(57.15)	107879317.32(58.22)	11642836.15(48.85)	
Former	52896302.75(25.29)	44612203.66(24.08)	8284099.08(34.76)	
Now	36711280.67(17.55)	32802562.39(17.70)	3908718.28(16.40)	
**Alcohol consumption**				0.003
No	145983953.37(69.81)	127710758.58(68.92)	18273194.79(76.66)	
Yes	63145783.52(30.19)	57583324.80(31.08)	5562458.72(23.34)	
**BMI**	29.81(0.28)	29.32(0.23)	33.62(0.66)	< 0.0001
**Weight status**				< 0.0001
Under/Normal weight	54863323.72(26.23)	52214289.98(28.18)	2649033.74(11.11)	
Overweight	63451153.38(30.34)	56825946.92(30.67)	6625206.46(27.80)	
Obese	90815259.79(43.43)	76253846.47(41.15)	14561413.32(61.09)	
**PHQ-9**				0.07
[0,4]	158017702.01(75.56)	141921761.47(76.59)	16095940.54(67.53)	
[5,9]	33643205.53(16.09)	28697715.62(15.49)	4945489.91(20.75)	
[10,14]	11457216.28(5.48)	9540025.82(5.15)	1917190.47(8.04)	
[15,19]	4646760.42(2.22)	3967541.71(2.14)	679218.71(2.85)	
[20,27]	1364852.65(0.65)	1167038.75(0.63)	197813.89(0.83)	
**PHQ-9 (cut-off by 5)**				0.03
No	158017702.01(75.56)	141921761.47(76.59)	16095940.54(67.53)	
Yes	51112034.88(24.44)	43372321.90(23.41)	7739712.98(32.47)	
**PHQ-9 (cut-off by 10)**				0.02
No	191660907.54(91.65)	170619477.10(92.08)	21041430.44(88.28)	
Yes	17468829.35(8.35)	14674606.28(7.92)	2794223.07(11.72)	
**Vigorous physical activity**				0.01
No	109876288.29(52.54)	95127108.95(51.34)	14749179.34(61.88)	
Yes	99253448.60(47.46)	90166974.43(48.66)	9086474.17(38.12)	
**Milk consumption**				0.13
Never	44514910.91(21.29)	38932674.27(21.01)	5582236.65(23.42)	
Rarely	46014509.91(22.00)	40194744.46(21.69)	5819765.45(24.42)	
Sometimes	60867854.33(29.11)	55517362.95(29.96)	5350491.39(22.45)	
Often	57732461.73(27.61)	50649301.70(27.33)	7083160.03(29.72)	
**DM**				< 0.001
No	163481295.44(78.17)	147719377.62(79.72)	15761917.82(66.13)	
IGT/IFG	13664270.94(6.53)	11623592.87(6.27)	2040678.07(8.56)	
DM	31984170.50(15.29)	25951112.88(14.01)	6033057.62(25.31)	
**Hyperlipidemia**				< 0.001
No	69225632.71(33.10)	63661385.13(34.36)	5564247.58(23.34)	
Yes	139904104.18(66.90)	121632698.25(65.64)	18271405.93(76.66)	
**Hypertension**				< 0.001
No	125474698.45(60.00)	115016544.32(62.07)	10458154.13(43.88)	
Yes	83655038.44(40.00)	70277539.06(37.93)	13377499.38(56.12)	
**Gallbladder surgery**				< 0.0001
No	183984464.26(87.98)	178896155.98(96.55)	5088308.28(21.35)	
Yes	25145272.63(12.02)	6397927.40(3.45)	18747345.23(78.65)	
**Missing teeth**	7.24(0.26)	6.87(0.26)	10.12(0.52)	< 0.0001
**Dietary fiber (g)**	16.86(0.43)	17.02(0.46)	15.66(0.50)	0.04
**Total fat (g)**	88.65(0.93)	89.75(0.97)	80.14(2.88)	0.01
**Cholesterol (mg)**	307.99(4.59)	313.75(4.60)	263.18(15.41)	0.004
**Glucose (mmol/L)**	5.53(0.04)	5.49(0.05)	5.82(0.09)	0.01
**Triglycerides (mmol/L)**	1.64(0.04)	1.62(0.04)	1.79(0.10)	0.04
**Total cholesterol (mmol/L)**	4.93(0.05)	4.93(0.05)	4.93(0.08)	1
**HDL-C (mmol/L)**	1.38(0.01)	1.38(0.01)	1.36(0.01)	0.09

PHQ-9: 9-question Patient Health Questionnaire; BMI: Body Mass Index; DM: Diabetes mellitus; IGT; impaired glucose tolerance; IFG: Impaired Fasting Glucose

**Table 2:** The association between missing teeth and self-reported gallstones

Missing teeth	Crude model		Model 1		Model 2		Model 3		Model 4	
	OR (95%CI)	P	OR (95%CI)	P	OR (95%CI)	P	OR (95%CI)	P	OR (95%CI)	P
T1	ref		ref		ref		ref		ref	
T2	2.75(1.95,3.87)	<0.0001	2.03(1.25,3.29)	0.01	2.03(1.22,3.36)	0.02	1.95(1.29,2.93)	0.003	1.94(1.29,2.91)	0.003
T3	3.63(2.57,5.13)	<0.0001	2.30(1.53,3.47)	0.001	2.24(1.41,3.55)	0.01	1.94(1.15,3.27)	0.02	1.93(1.14,3.26)	0.02
p for trend		<0.0001		<0.001		0.003		0.01		0.01

Crude model: Unadjusted model Model 1: Adjusted for age, sex and ethnic Model 2: Additionally adjusted for marital status, educational level, and Family monthly poverty level Model 3: Additionally adjusted for smoking, alcohol consumption, BMI, PHQ-9, Vigorous physical activity, and milk consumption Model 4: Additionally adjusted for dietary fiber, total fat, and cholesterol OR: odds ratios; CI: confidence interval

**Table 3:** Stratified analysis of self-reported gallstones across missing teeth

Character	T1	T2 (OR [95% CI])	P value	T3 (OR [95% CI])	P value	p for trend	P for interaction
**Age group**							0.72
**20-40**	ref	2.26(1.01, 5.06)	0.05	4.75(0.90,25.07)	0.06	0.02	
**40-60**	ref	1.71(0.81,3.60)	0.15	1.84(0.96,3.51)	0.06	0.06	
**≥60**	ref	2.03(1.03,3.97)	0.04	1.86(0.84,4.16)	0.12	0.17	
**Sex**							0.42
**Female**	ref	1.81(1.22,2.68)	0.01	2.07(1.27,3.37)	0.01	0.005	
**Male**	ref	2.42(0.87, 6.77)	0.09	1.88(0.79, 4.46)	0.14	0.1	
**ethnic**							0.7
**White**	ref	1.98(1.13,3.45)	0.02	1.97(1.11,3.51)	0.02	0.02	
**Black**	ref	0.98(0.29, 3.29)	0.97	2.31(0.66, 8.08)	0.17	0.16	
**Other**	ref	2.11(1.06, 4.20)	0.04	1.91(0.98, 3.72)	0.06	0.04	
**Educational level**							0.86
**≤Highschool**	ref	1.60(0.76,3.35)	0.2	1.51(0.72,3.17)	0.25	0.29	
**>Highschool**	ref	2.26(1.45,3.51)	0.001	2.35(1.28,4.30)	0.01	0.003	
**Marital status**							0.49
**Alone**	ref	2.21(1.01, 4.85)	0.05	1.96(0.86, 4.45)	0.1	0.1	
**Married/With partner**	ref	1.87(0.96,3.62)	0.06	1.99(1.10,3.57)	0.02	0.02	
**Family monthly poverty level**							0.3
**≤1.30**	ref	1.19(0.63, 2.25)	0.58	1.57(0.67, 3.67)	0.28	0.28	
**1.30-1.85**	ref	1.06(0.34, 3.26)	0.92	1.28(0.45, 3.60)	0.62	0.64	
**>1.85**	ref	2.45(1.41,4.25)	0.003	2.32(1.15,4.68)	0.02	0.01	
**Smoking**							0.54
**No**	ref	2.19(1.29,3.71)	0.01	1.82(0.96,3.46)	0.07	0.01	
**Yes**	ref	1.72(1.03,2.84)	0.04	2.12(1.22,3.67)	0.01	0.01	
**Alcohol consumption**							0.84
**No**	ref	2.20(1.21,4.02)	0.01	2.21(1.36,3.60)	0.003	0.003	
**Yes**	ref	1.36(0.49, 3.75)	0.53	1.71(0.42, 7.05)	0.43	0.43	
**Weight status**							0.31
**Under/Normal weight**	ref	2.75(0.68,11.21)	0.15	2.72(0.75, 9.87)	0.12	0.08	
**Overweight**	ref	1.77(0.62, 5.07)	0.26	2.28(0.83, 6.23)	0.1	0.11	
**Obese**	ref	1.95(1.12,3.38)	0.02	1.76(1.17,2.64)	0.01	0.01	

OR: odds ratios; CI: confidence interval Adjusted for age, sex, ethnic, marital status, educational level, Family monthly poverty level, smoking, alcohol consumption, BMI, PHQ-9, Vigorous physical activity, milk consumption, dietary fiber, total fat, and cholesterol

**Table 4:** Mediation effect of the age and BMI on the association between missing teeth and self-reported gallstones

	BMI (95% CI)	P value	Glucose (95% CI)	P value	Triglycerides (95% CI)	P value	Total cholesterol (95% CI)	P value	HDL-C (95% CI)	P value
**Direct effect**	0.02(0,0.04)	< 0.0001	0.02(0,0.05)	0.02	0.02(0,0.05)	< 0.0001	0.02(0,0.05)	0.06	0.02(0,0.04)	< 0.0001
**Mediated (indirect) effect**	0(0,0)	0.78	0(0,0)	0.02	0(0,0)	0.16	0(0,0)	0.7	0(0,0.01)	0.06
**Total effect**	0.02(0,0.04)	< 0.0001	0.02(0,0.05)	< 0.0001	0.02(0.01,0.05)	< 0.0001	0.02(0,0.05)	0.06	0.02(0,0.04)	0.04
**Proportion mediated (%)**	1.59		4.91		3.05		0.9		11.97	

Adjusted for age, sex, ethnic, marital status, educational level, Family monthly poverty level, smoking, alcohol consumption, BMI, PHQ-9, Vigorous physical activity BMI: Body Mass Index; HDL-C: High Density Lipoprotein Cholesterol; CI: confidence interval;

## Abbreviations

BMI: body mass index
NHANES: National Health and Nutrition Examination Survey
PHQ-9: 9-question Patient Health Questionnaire
OR: odds ratio
CI: confidence interval
