# Supplementary material for: Association of tooth loss and gallstones: National Health and Nutrition Examination Survey 2017-2018
Source: Int J Med Sci. 2024 Jul 16;21(10):1866–75. doi: 10.7150/ijms.98492 (PMC11302568; doi:10.7150/ijms.98492)
Supplement: Supplementary file 1 — Supplementary table. [file ijmsv21p1866s1.pdf]

Caption for supplementary material

Appendix 1. Supplementary Tables

Appendix 1. Supplementary tables

Table S1. The association between bone loss, root caries, gum disease, and self-reported gallstones

Variables	Crude model		Model 1		Model 2		Model 3		Model 4	
	OR (95%CI)	P	OR (95%CI)	P	OR (95%CI)	P	OR (95%CI)	P	OR (95%CI)	P
**Bone loss**										
No	ref		ref		ref		ref		ref	
Yes	2.19(1.54,3.12)	<0.001	1.87(1.34,2.61)	0.002	1.84(1.27,2.65)	0.01	1.76(1.27,2.43)	0.002	1.78(1.27,2.48)	0.002
**Root caries**										
No	ref		ref		ref		ref		ref	
Yes	1.79(1.22,2.62)	0.01	1.83(1.17,2.87)	0.01	1.74(1.02,2.98)	0.04	1.68(0.98,2.87)	0.06	1.66(0.96,2.87)	0.07
**Gum disease**										
No	ref		ref		ref		ref		ref	
Yes	1.31(0.95,1.81)	0.09	1.45(0.97,2.15)	0.07	1.41(0.91,2.21)	0.10	1.31(0.84,2.07)	0.22	1.31(0.83,2.04)	0.22

Crude model: Unadjusted model

model 1: Adjusted for age, sex and ethnic

model 2: Additionally adjusted for marital status, educational level, and Family monthly poverty level

model 3: Additionally adjusted for smoking, alcohol consumption, BMI, PHQ-9, Vigorous physical activity, and milk consumption

model 4: Additionally adjusted for dietary fiber, total fat, and cholesterol

OR: odds ratios; CI: confidence interval;
